# Difficulties and countermeasures in implementing age-friendly policies in primary health centers in Taiwan

**DOI:** 10.1186/s12913-022-08770-0

**Published:** 2022-11-14

**Authors:** Chen-I Shih, Tuey-Wen Hung, Wei Chen, Hui-Fei Yang, Shu-Li Chia, Yung-Hung Chang, Sheng-Yu Fan

**Affiliations:** 1 Department of Community Health, Ditmanson Medical Foundation Chia-Yi Christian Hospital, Chia-Yi, Taiwan; 2 Department of Family Medicine, Ditmanson Medical Foundation Chia-Yi Christian Hospital, Chia-Yi, Taiwan; 3 Medical Department, Ditmanson Medical Foundation Chia-Yi Christian Hospital, Chia-Yi, Taiwan; 4 Health Promotion Administration, Ministry of Health and Welfare, Taipei, Taiwan; 5 Institute of Gerontology, College of Medicine, National Cheng Kung University, No.1, University Road, Tainan City, 701 Taiwan

**Keywords:** Age-friendly, Primary health centers, Quality management

## Abstract

**Background:**

Taiwan is predicted to become a super-aged society by 2025, and primary health centers (PHCs) are set to play a crucial role in the care of older adults. The Taiwanese government has developed an age-friendly verification framework for PHC. The aims of this study were to explore the difficulties faced by PHC staff in the implementation of age-friendly policies and their solution strategies.

**Methods:**

This study adopted a qualitative research method. The first stage involved conducting five focus groups with the responsible staff of PHCs (*n* = 41) that have been certified “age-friendly.” The focus groups covered the effectiveness, difficulties, and resources of PHCs in regards to the introduction of age-friendly policies. In the second stage, in-depth interviews were conducted with executives of PHCs (*n* = 5), both certified and not certified as age-friendly, to further compare the difficulties faced by these two types of PHCs, thereby gaining perspectives for solution strategies. The principles of grounded theory were used for data analysis.

**Results:**

Four major PHC strategies are employed in the promotion of age-friendliness. First, organizational management, through which managers apply management methods and analyze the present PHC-related health concerns; second, resource utilization, which refers to the tallying, linking, and integrating of resources; third, business operation process, in which work efficiency is improved through the combination of business operations and staff training; finally, hardware improvement, which is achieved through comprehensive cataloging of facility environments.

**Conclusion:**

The implementation of age-friendliness in PHCs requires the efforts of both the service units and government. With resources provided by the government, PHCs can integrate management methods, businesses operations, and essential resources. Moreover, PHC executives can lead their teams in promoting age-friendly policies, and closely monitor their effectiveness.

## Background

Population aging is a global trend. According to the State of World Population Report, older adults will account for 16% of the world’s population by 2050 [1]. Taiwan’s older population exceeded 14% in 2018, and this proportion is expected to exceed 20% by 2025 [2]. Primary health centers (PHCs) are the fundamental community health service in Taiwan’s health care system. PHCs provide crucial health services that include community health care, family health management, and individual care [3–5].

In response to health care issues arising from an aging society, the Taiwanese government developed an age-friendly certification framework for PHCs in 2016 [6]. The certification comprises the following five standards: (1) Management policy: PHCs are required to establish age-friendly policies and resources; (2) Information intervention and communication: Staff are provided with age-friendly training, can adjust administrative procedures, and engage in shared decision-making (SDM) with older adults; (3) Friendly environment: PHCs are required to provide a barrier-free, healthy, and healing environment that satisfies the needs of older adults; (4) Health promotion: Older adults are assessed and individual care plans are developed accordingly; and (5) Community services and referrals: Community resources are evaluated and integrated for the provision of resources or referrals [6].

Between 2017 and 2019, 322 PHCs in Taiwan were granted age-friendly certification. These PHCs exhibited superior performance in the establishment of age-friendly policies and resources as well as the integration of community resources for enhanced services. In addition, staffs receive age-friendly training and are able to adjust service procedures. However, some PHCs underperformed in regards to the application of management methods, promotion of health awareness, SDM and encountered difficulties in establishing an age-friendly environment [7].

International studies have reported that PHCs require cooperation across various professional teams to provide age-friendly services to the community. Thus, leaders are required to organize teams, assign tasks, develop community cooperation networks, connect medical and social services, and provide quality geriatric care [8, 9]. At present, PHCs encounter the following problems: general practitioners are faced with an excessive workload, lack of funds and are not motivated to lead a transdisciplinary team [8, 9]. To exacerbate matters, public health nurses largely focus on personal care, and their excessive workload renders them unable to address wider-scale population health [10, 11].

The Taiwanese government proactively promotes the introduction of age-friendly policies to PHCs, but PHC staff may encounter difficulties during the certification process. A comprehensive assessment of PHCs’ implementation of age-friendly policies from the perspective of service providers was undertaken. Therefore, by conducting focus groups and interviews, we aimed to obtain an in-depth understanding of the experiences and difficulties encountered by PHC staff in the implementing of age-friendly policies, as well as solution strategies they used.

## Method

### Research design and participants

This study was conducted based on the principles of grounded theory [12], and applied a two-stage qualitative research methodology, wherein the first stage involved focus groups and the second stage individual in-depth interviews. The second stage was designed in case the focus group participants could not fully express their thoughts in the first stage. Taiwan government has developed an age-friendly certification framework for PHCs in 2016 [6] and then applied the framework to evaluate whether PHCs met the criteria of age-friendly certification. The decision was made by a healthcare professionals committee based on the framework. By comparing PHCs certified and uncertified as age-friendly, we further explored the problems observed in the focus group, centering on solution strategies.

In research participants, we conducted purposive sampling. In the first stage of this qualitative research, we conducted focus groups with executives of PHCs that had gained age-friendly certification and had been implementing age-friendly policies for longer than 5 months, or with those in charge of the PHCs’ age-friendly services. In the second stage, we held in-depth interviews with PHC directors or head nurses from PHCs both certified and uncertified as age-friendly.

Before implementation, this research project was approved by a hospital’s institutional review board about research ethics approval (IRB No.: CYCH-IRB2018030). We informed the participants of their rights and obligations prior to data collection. Participants were ensured of their anonymity during the audio recording of the interview and were informed of their right to withdraw from the study at any time. Participants provided written informed consent before their participation, and each received NT$2000 after completion as rewards.

### Data collection

The focus groups covered the following topics: (1) The changes made by PHCs to introduce age-friendly policies; (2) The effectiveness of the management methods employed in PHCs; (3) The willingness of cooperating community organizations to comply with age-friendly policies; (4) Examples of the achievements and difficulties encountered in promoting age-friendliness; and (5) The changes made by PHCs to cultivate an age-friendly environment.

The initial analysis of the focus group transcripts was used as the basis for the development of the individual interview guidelines to enable comprehensive comparison of problems and propose solutions. The outline comprised the following questions on difficulties related to age-friendly initiatives, which were followed by a question asking how to overcome these difficulties: (1) What are the difficulties in assessing the issues of older adults in the community? (2) What are the difficulties in applying management methods? (3) What are the difficulties in linking and integrating resources? (4) What are the difficulties in cultivating an age-friendly environment? and (5) Is there any issues on staff workload? Some probe questions were also used to explore the further meanings, for example the difficulties, internal and external resources, and the specific needs for execution of age-friendly policies. Prior to the focus groups and interviews, the guidelines were reviewed and revised by three professors from the Department of Public Health, Institute of Gerontology, and Department of Sociology, respectively, and a senior PHC director.

All focus groups were moderated by the same leader who is a professor in public health and had participated in the development of PHC age-friendly frameworks. All in-depth interviews were conducted by the first author, who had participated in the on-site certification of PHC age-friendly frameworks and had received interviewer training.

### Data analysis

This study employed grounded theory for data analysis [12], which consisted of the following steps: (1) Open coding: identify and conceptualize keywords in transcripts; categorize concepts under the name of a higher-level concept. For example, the response “circular quality management is necessary” was coded as “quality management” along with other relevant statements and grouped into the same category under the concept “management method;” (2) Axial coding: through contextual analysis and identifying connections across categories, data were recombined to construct “axes.” For example, aggregate three categories (i.e., problem analysis, executive leadership, and management method) under the theme entitled “organizational management;” (3) Selective coding: construct a core category to explain the relationship between different aspects of a narrative. For example, forming a core category with organizational management, resource utilization, business operation process, and hardware improvement.

The first author conducted the initial coding, and the corresponding authors reviewed the coding for internal consistency; all authors integrated multiple data from focus groups, interviews, and field notes to achieve the final themes. The data collection and analysis process were conducted in Chinese and the final manuscript was writing in English. We conducted analysis whilst data was still being collected, and the collection of samples continued until data saturation was reached and no new themes emerged [13].

This study applied the following four methods to ensure rigorousness in the qualitative research: (1) Credibility: The research team included professional geriatric case managers, physicians, and clinical psychologists, with rich experience in geriatric care, primary care, and conducting qualitative research and interviews; (2) Transferability: Interviewers repeatedly verify data with the interviewee during the interview and examine the data’s relevance to the research questions during analysis; (3) Confirmability: The first author conducted the initial coding and other authors recheck to ensure internal consistency. In addition, the completed transcripts are sent to some interviewees (*n* = 5) for confirmation of the transcribed content; (4) Dependability: To avoid errors produced by conducting an excessive number of interviewers, one leader conducted all focus groups and one interviewer conducted all in-depth interviews. The qualitative analysis software Altasi.6.0 was used to categorize and summarize the collected data. The Standards for Reporting Qualitative Research (SRQR) was used to report this study [14].

### Results

Five focus groups were held. Each group was attended by 6 to 10 interviewees and lasted 2.5 to 3 hours. A total of 41 PHC staff took participation, including 13 directors, 19 head nurses, and 9 people in charge of age-friendly services (i.e., nurse practitioners). Four PHC directors and one head nurse were invited to participate in the in-depth interviews. Two of the PHC directors also engaged in the focus groups. Each in-depth interview lasted 1 to 1.5 hours. Descriptive statistics of the demographics of the focus group and in-depth interview participants are listed in Table 1.

**Table 1:** Descriptive statistics of the demographics of the focus group and in-depth interview participants

Item	Focus group (*N* = 41)	In-depth interview (*N* = 5)
**Gender**		
**Woman**	33	2
**Man**	8	3
Position at the PHC		
Director	13	4
Head nurse	19	1
Staff of age-friendly services	9	0
PHC location		
Northern Taiwan	15	3
Central Taiwan	9	1
Southern Taiwan	9	0
Eastern Taiwan	4	1
Outer islands	4	0

Four themes were identified, including organizational management, resource utilization, business operation process, and hardware improvement, with the relevant information detailed in Table 2.

**Table 2:** Overview of the topics and subtopics

Theme	Subtheme
Organizational management	Problem analysis
	Executive leadership
	Management method
Resource utilization	Resource inventory
	Resource linkage
	Resource integration
Business operation process	Business consolidation
	Establishment of a standardized operating procedure
	Employee training
Hardware improvement	Gradual improvement
	Self-design
	Alternative improvement plan

#### Theme 1: organizational management

Participants observed that PHCs have rarely managed age-friendly policies in a systematic manner. Therefore, PHC directors are required to use strategies on problem analysis, executive leadership and management methods to effectively conduct organizational management. Regarding problem analysis, PHC directors shall first assess the health problems and needs of the community’s older adults, such as the prevalence of chronic diseases. They can then apply management strategies to review their internal and external advantages and disadvantages of PHCs before formulating a plan that meets the needs of older adults  .

*Focus group case 28: In reference to the top ten causes of death … , diabetes should be addressed as our first priority. Therefore, we established a support group for diabetic patients. (Director in Southern Taiwan)*

*In-depth interview case 4: We conducted outpatient questionnaire surveys to understand local problems... SWOT [strengths, weaknesses, opportunities, and threats] analysis should be performed first to identify our internal and external strengths and weaknesses. (Director in Central Taiwan)*

In terms of executive leadership, directors stated that they may first build consensus among staff to determine their shared vision, thereby enabling staff to voluntarily participate in the age-friendly works. Moreover, directors can provide support and resources for staff to effectively implement age-friendly plans.

*Focus group case 37: Communication is extremely crucial. Only by reaching a consensus can colleagues accept [the concept of age-friendliness] and develop a shared mission. (Head nurse in Eastern Taiwan)*

*Focus group case 25: The support from the executive has united all the staff... The most important thing is to introduce these concepts to colleagues, and the executive has allowed us to do so by actively arranging visits [to other PHCs to learn from them]. (Nurse practitioner in Southern Taiwan)*

In regards to management methods, PHC directors can undertake continuous monitoring through the application of the appropriate quality management method. PHC directors may establish evaluation indicators, revise relevant operating standards in relation to their efficacy, and analyze the reasons for the inferior effect of certain items to implement corrective measures.

*In-depth interview case 1: Data from strategy maps, KPIs [key performance indicators], the lean management approach, and fishbone diagrams are integrated. PDCA [Plan-Do-Check-Act] is a must and should be repeated constantly to identify problems; calibration should be redone every year. (Director in Northern Taiwan)*

#### Theme 2: resource utilization

PHCs have limited budgets, and the subsidies they receive are sometimes insufficient to implement all the steps required for age-friendly certification. Therefore, before implementing age-friendly plans, PHC directors suggest to perform an inventory of internal and external resources and integrate external resources as required. An annual review of the resources available to PHCs can facilitate the subsequent planning of an age-friendly environment, and formulation of an annual plan for community-based geriatric health care services.

*In-depth interview case 2: We will inventory all the available resources, including external community resources and internal resources, be it projects or external funds...We need to allot time and examine this year’s budget before deciding what can be achieved this year. (Director in Northern Taiwan)*

Regarding resource linkage, PHCs can connect resources to apply them to areas requiring improvement. First, PHCs can emphasize the essentials of age-friendliness to local political and community association leaders and request resources and support to strengthen the effective execution of age-friendly policies. Subsequently, PHCs can cooperate with communities to build a resource network, thereby providing older adult with diverse services.

*In-depth interview case 4: Raise funds as needed … request Lions Clubs International [funding] for ophthalmic equipment. In addition, Huashan Social Welfare Foundation, local philanthropists, Red Cross Society of the Republic of China … all expressed their willingness to support the campaign. (Director in Central Taiwan)*

After undertaking resource inventory and linkage, PHCs can proceed with resource integration. Efforts shall be made to continuously strengthen the connection and integration of community resources while merging and managing the internal resources of PHCs. Internal and external resources are used in combination to gradually cultivate age-friendliness in PHCs.

*Focus group case 21: After gathering together all the resources available, we applied for these projects. Some funds can be used flexibly for integration with other funds. (Head nurse in Central Taiwan)*

#### Theme 3: business operation process

PHCs provide a wide range of services, which impose a heavy workload on staff. To implement age-friendly plans as scheduled, PHC staff suggest to incorporate age-friendliness into other business frameworks and offer age-friendly staff training. In terms of business consolidation, PHCs can simplify operating procedures and projects to use time efficiently. In addition, different projects of the same business type could be merged to reduce staff work efforts being squandered on repetitive tasks.

*In-depth interview case 4: Never waste time on repeated transportation routes when scheduling visits … You have failed to manage your time if you feel busy or need to work overtime. (Director in Central Taiwan)*

*Focus group case 11: Plans can be combined...For example, the integration of community care with long-term care and age-friendliness can reduce the workload of PHC staff... The obtained results can be applied extensively. (Head nurse in Northern Taiwan)*

For operating procedures, PHCs can formulate or simplify standardized work processes as required, conduct knowledge management, and establish information management platforms (e.g., public folders). These actions would facilitate the storage, transmission, consultation, and use of knowledge for PHC staff, as well as assisting with experience sharing, staff learning, and goal achievement in relation to age-friendliness through knowledge integration.

*Focus group case 37: We have simplified the procedure for long-term care application considering that the area occupied by our tribe is relatively narrow and long. (Head nurse in Eastern Taiwan)*

In terms of staff training, age-friendly education and training should be provided. Both executives and staff could strengthen their ability to identify and solve problems, thereby enhancing business consolidation skills and reducing workload. In addition, age-friendly training would serve to broaden their knowledge of older adults.

*In-depth interview case 5: The concept of SWOT has been recognized by the junior staff and can be applied in their work. (Director in Eastern Taiwan)*

#### Theme 4: hardware improvement

PHC hardware is seldom updated because of their old building structures and insufficient funds. However, PHCs can still allocate funds annually for self-repair and alternative improvement plans. In regards to the gradual improvement of equipment, PHCs are largely housed in old buildings with insufficient budgets for maintenance. Therefore, PHCs are advised to plan and integrate expenditure, beginning with small expenses, to allocate funds for equipment improvement. The objective is to meet the basic needs of older adults, with minor renovations at the outset and gradual refinement of an age-friendly environment year by year.

*Focus group case 12: When facing the problem of insufficient funds for the renovation of old buildings, a list of priority items requiring improvement should be compiled to achieve a balance between the aspects of public budget and hospital services. You can still be economical with NT$100,000 by beginning with the smallest refurbishment to meet public needs. The basic structure can be gradually improved and refined later. (Director in Northern Taiwan)*

With regard to self-repair, PHCs can first conduct a comprehensive inspection from the perspective of older adults. That is, the experience of older adults from the moment they enter the PHC to when they leave after receiving the service. Improvements are to be made to smooth the obstacles encountered by older adults during the process. In the event of a budget deficit, PHCs may complete simple do-it-yourself repairs using purchased materials (e.g., nonslip strips for the floor).

*Focus group case 7: Comprehensively examine the process and environment of service provision from the perspective of older adults to identify necessary improvements. (Head nurse in Northern Taiwan)*

*Focus group case 5: Install self-made handrails next to the scales and ask older adults to test them out. Purchase nonslip strips and apply them to stairs, instead of getting it done by others. (Head nurse in northern Taiwan)*

In terms of alternative improvement plans, no drastic changes can be made to building structures because many PHCs share their building with other public departments, or are simply too old for refurbishment. This problem can be resolved through the establishment of alternative improvement plans, such as the manual provision of mobile services to reduce the environmental barriers for older adults and enhance convenience.

*Focus group case 41: In response to outdated hardware, we have appointed designated people to provide mobile services. Age-friendly counters have also been designated to cater to the needs of older adults as soon as they enter the PHC. If older adults need a blood test, this can be performed without them getting off their mobility scooters. (Head nurse in the outer islands)*

## Discussion

This study applied a qualitative method to explore the difficulties encountered by PHCs in promoting age-friendly policies and establishing solution strategies. Organizational management, resource utilization, business processes, and hardware improvement are crucial for cultivating age-friendliness in PHCs. In addition to problem analysis and resource inventory and integration, PHCs should merge overlapping work, optimize the operating process, and provide staff training. In terms of hardware, PHCs can gradually allocate funds for self-repair and design alternative plans to improve the hardware environment.

Informed planning is necessary in PHCs’ formulation of age-friendly initiatives. That is, PHC directors can first investigate the overall needs of the local older adults to understand their problems and set clear goals. In addition, PHCs can apply strategic management methods (e.g., SWOT) for analyzing external opportunities and problems; identifying internal strengths and weaknesses; and selecting the most appropriate solution strategy according to the goals of their age-friendly plans [15]. The implementation of age-friendly policies requires PHCs to incorporate the concept of management [16]. In respect to planning, PHCs have to determine set goals and methods for the practice of age-friendly business. As part of their organizational and leadership strategies, PHCs should effectively allocate their workforce and related resources and motivate their employees to achieve their goals. In terms of control, PHCs should closely monitor performance execution [17, 18].

PHCs can prepare the necessary resources for age-friendly plans. First, PHC directors can obtain an inventory of available resources in accordance with the goals of their age-friendly plan. PHCs’ internal resources include their workforce, hardware, funds, information, and technical resources [18], and their external resources refer to both tangible (e.g., human, financial, and material resources) and intangible resources (e.g., social awareness and other humanistic resources) [19]. Second, PHC directors can link resources according to their plan requirements through emphasizing the necessity of age-friendliness to local leaders. Cooperation with communities is necessary for the joint establishment of a resource network [20]. Third, PHC directors can combine internal, external, tangible, and intangible resources to facilitate communication and establish an operational mechanism for integrating resources [18]. According to the needs of the community’s older adults, PHCs can formulate resource management plans and then monitor age-friendly indicators for regular performance assessment [18, 21].

PHCs can also integrate business operation processes. PHC directors can use a flow chart to determine the time afforded to each step of the care services for older adults. Repeated or unnecessary steps are then removed, and the smooth operation of the necessary steps is ensured [22, 23]. Following business consolidation, PHC directors may establish standard operating procedures to ensure that age-friendly services can be effectively provided using the same approach with minimal time consumption, thereby maintaining the take time [22, 24]. Knowledge management, another requirement, can be achieved through the establishment of a data-sharing platform for integrating and transmitting knowledge; this maintains the effectiveness of PHCs’ implementation of age-friendly business and culture within the organization [25, 26]. Furthermore, directors and staff ought to receive education and training that focus on the goal of age-friendliness, identifying the problems experienced by older adult and proposing solutions, utilizing available resources and technologies, and satisfying employees’ learning needs (e.g., business consolidation and training for geriatric care) [18]. In terms of hardware improvement, PHCs can integrate funds and formulate a repair plan; following the gradual improvement of hardware, PHCs can conduct regular assessments of the results [27]. For repair planning, PHCs can begin undertaking small-scale self-repairs, such as installing a service bell at the entrance. If refurbishment is not an option, PHCs may consider developing alternative plans. For example, PHC staff could manually assist older adults up and down steep ramps [28, 29].

PHC directors should also guide employees to achieve the goals of their age-friendly plans. In terms of leadership, PHC directors can outline a clear age-friendly vision to motivate employees, allowing employees to learn from each other, effectively communicate with others, and reach consensus. The systems, job positions, and organizational structure of the team in charge of relevant businesses should be clearly defined to ensure that age-friendly plans can be implemented [6, 30]. Lastly, PHC directors can control the development of the age-friendly plans through the utilization of quality management methods (e.g., PDCA) to ensure the achievement of goals and promote the continual monitoring and improvement of service quality [18].

An aging society requires PHCs to establish a multidisciplinary primary health care team for geriatric care. Because PHCs provide a wide range of services, the literature has reported problems with insufficient resources, business overlap, and workforce shortages in PHCs, which deprives the centers of time that could be spent serving as community health care promoters [3, 10, 31]. In response, the Taiwanese government relaxed restrictions on the number of medical staff a PHC may hire to increase personnel, advocated for a hub plan, formed a community-based inter unit network for the integration of age-friendly resources, promoted construction projects, and subsidized PHC funds to repair barrier-free equipment.

In this study, we used qualitative methodology to gain insight into the management problems facing PHC executives when promoting age-friendly plans and proposed feasible solutions. However, the data was from mangers rather than employee. They may have different perspectives about work loading, difficulties, and strategies about age-friendly plans. In addition, the perspectives from older adults and families are necessary for this issue. Perspectives from multiple sources can be addressed in the future studies.

## Conclusion

The greatest difficulties facing PHCs in the promotion of age-friendly policies are business overlap, insufficient funds, and workforce shortages. The implementation of age-friendly policies requires not only the government to provide adequate funding and human resources, but also the highly-skilled management of PHC directors. With these factors, problem analysis can be undertaken to resolve geriatric issues, resource tallying and integration can be performed according to specific research objectives, and PHC software and hardware can be gradually repaired. When implementing age-friendly plans, PHC directors are advised to merge overlapping businesses, establish standard operating procedures, and provide employee training to enhance effectiveness. Furthermore, they may apply management methods for quality monitoring to continuously optimize the provision of age-friendly services.

## Data Availability

All data was reported in this study, and additional data are not available due to data protection restrictions.
